# Advancing screening tool for hospice needs and end-of-life decision-making process in the emergency department

**DOI:** 10.1186/s12904-024-01391-w

**Published:** 2024-02-23

**Authors:** Yu-Jing Wang, Chen-Yang Hsu, Amy Ming-Fang Yen, Hsiu-Hsi Chen, Chao-Chih Lai

**Affiliations:** 1 Department of Emergency Medicine, Taipei City Hospital, Taiwan. No. 10, Sec. 4, Ren-Ai Road, Ren-Ai Branch, Taipei, Taiwan; 2 Master of Public Health Program, National Taiwan University, Taipei, Taiwan; 3 Medical Department, Daichung Hospital, Miaoli, Taiwan; 4 Taiwan Association of Medical Screening, Taipei, Taiwan; 5 School of Oral Hygiene, College of Oral Medicine, Taipei Medical University, Taipei, Taiwan; 6 Institute of Epidemiology and Preventive Medicine, College of Public Health, National Taiwan University, Taipei, Taiwan

**Keywords:** Emergency department, Palliative care, Hospice care, Prognosis, End of life care, Resuscitation orders, Retrospective study, Physical performance, Decision-making

## Abstract

**Background:**

Predicting mortality in the emergency department (ED) is imperative to guide palliative care and end-of-life decisions. However, the clinical usefulness of utilizing the existing screening tools still leaves something to be desired.

**Methods:**

We advanced the screening tool with the A-qCPR (Age, qSOFA (quick sepsis-related organ failure assessment), cancer, Performance Status Scale, and DNR (Do-Not-Resuscitate) risk score model for predicting one-year mortality in the emergency department of Taipei City Hospital of Taiwan with the potential of hospice need and evaluated its performance compared with the existing screening model. We adopted a large retrospective cohort in conjunction with in-time (the trained and the holdout validation cohort) for the development of the A-qCPR model and out-of-time validation sample for external validation and model robustness to variation with the calendar year.

**Results:**

A total of 10,474 patients were enrolled in the training cohort and 33,182 patients for external validation. Significant risk scores included age (0.05 per year), qSOFA ≥ 2 (4), Cancer (5), Eastern Cooperative Oncology Group (ECOG) Performance Status score ≥ 2 (2), and DNR status (2). One-year mortality rates were 13.6% for low (score ≦ 3 points), 29.9% for medium (3 < Score ≦ 9 points), and 47.1% for high categories (Score > 9 points). The AUROC curve for the in-time validation sample was 0.76 (0.74–0.78). However, the corresponding figure was slightly shrunk to 0.69 (0.69–0.70) based on out-of-time validation. The accuracy with our newly developed A-qCPR model was better than those existing tools including 0.57 (0.56–0.57) by using SQ (surprise question), 0.54 (0.54–0.54) by using qSOFA, and 0.59 (0.59–0.59) by using ECOG performance status score. Applying the A-qCPR model to emergency departments since 2017 has led to a year-on-year increase in the proportion of patients or their families signing DNR documents, which had not been affected by the COVID-19 pandemic.

**Conclusions:**

The A-qCPR model is not only effective in predicting one-year mortality but also in identifying hospice needs. Advancing the screening tool that has been widely used for hospice in various scenarios is particularly helpful for facilitating the end-of-life decision-making process in the ED.

**Supplementary Information:**

The online version contains supplementary material available at 10.1186/s12904-024-01391-w.

## Introduction

Early identification of nearing the end-of-life (EOL) can aid in identifying and addressing palliative care (PC) needs in the emergency department (ED) [1–8]. This is particularly important for older and seriously ill patients, who may benefit greatly from PC referrals. Such referrals can provide high-quality support and reduce suffering at the EOL [5]. Early identification of unmet PC needs and the provision of effective and efficient PC in the ED may improve the quality of EOL support, enhance the satisfaction of patients and their families, optimize the outcomes related to hospice care, and reduce the need for subsequent ED care and cost saving [5, 9, 10]. 

Current screening tools available for identifying patients with advanced progressive disease who may require PC in primary care settings are rather limited [11]. Accurate estimation of prognosis is informative to provide an optimal plan for treatment and EOL care in patients with advanced cancer.^10,11^ It can also relieve patient and career anxiety about the uncertainty of prognosis.^12^ The surprise question (SQ) is widely used for predicting mortality within 6 months. However, there was a wide degree of accuracy (0.51 to 0.82), and it seems subjective and more accurate in an oncology setting than others [12]. Though the functional status assessment tools, such as Karnofsky Performance Score, and the simplified Eastern Cooperative Oncology Group (ECOG) Performance Score, are widely used for screening, they lack accurate predicting ability or some are limited to advanced cancer in past reports.^13–15^ However, the ability of current screening tools to identify PC needs in primary care was still limited in the previous study [11]. Non-oncologist or non-hospice specialists cannot early identify the EOL and initiate PC. It should be noted that the previous study has shown a clinician-oriented communication strategy significantly bolstered the documentation of care objectives in hospitalized elderly patients with severe conditions [13]. 

In recent years, ED providers have long recognized the importance of assessing patients for hospice eligibility. However, there are no standardized criteria because these criteria and tools have not yet been standardized for ED patients. Screening patients for PC referrals and resources by developing a mortality prediction model in the ED is a feasible approach and may yield significant benefits [14]. A comprehensive analysis found that 53.4% of Medicare hospice beneficiaries had a high demand for PC before death, due to a tremendous increase in individuals diagnosed with non-malignant conditions, who were twice as likely to have these enrolment patterns as their oncology counterparts [15]. This implies that the development of the mortality prediction model needs to consider the robustness of the model to the enrolment pattern and types of patients with potential hospice needs, which has barely been addressed.

The model was developed for predicting one-year mortality in the emergency department, and it had high sensitivity and high accuracy of prediction in our previous preliminary report [16]. The model termed the A-qCPR model, incorporates factors such as age, qSOFA, cancer, performance status scale, and DNR. A larger cohort is required to validate its clinical usefulness to establish a standardized screening process that can not only predict mortality and deterioration but also anticipate PC needs and forecast the rate and trajectory of functional decline. Therefore, given the scenario of patients enrolled from the ED, our study aims are to improve the accuracy of the proposed A-qCPR risk model for predicting one-year mortality, to advance this screening tool for early identification of hospice needs, and to aid EOL decision-making.

## Methods

### Study population

Study subjects were enrolled from a retrospective cohort on patients aged 15 years or older who were admitted from the emergency department of Taipei City Hospital between 2015 and 2020. Patients younger than 15 years of age were excluded in this study. Patients younger than 15 years of age were excluded in this study. Taipei City Hospitals a multi-faceted healthcare organization with several branches scattered throughout Taipei, Taiwan. The locations of Taipei City Hospital’s branches are shown in Figure S1. It is composed of five branches of the hospital include Heping Fuyou, Renai, Zhongxing, Zhongxiao, and Yangming. These branches provide a wide range of medical services including PC. The details of medical service provided from Taipei City Hospital refer to Appendix. The relevant data from Taipei City Hospital are also shown in Table S1. There are a total of 55,735 (Standard deviation = 7006.5) annual average visits from five branches. All ED patients were collected from general branches of Taipei City Hospital in this study.

### Data collection

The automated risk scoring system was established at the end of 2017 after our first preliminary report because it was highly accurate for the cross-validation model [16]. Our ED patients were stratified into low, medium, or high mortality risk groups according to the model, which is displayed in the list of our HIS (hospital information system). This information is disseminated to all medical staff within the emergency department. It is used for family consultations and patient-physician interactions. We have not established specific responses to this risk classification; it does not influence or interfere with subsequent medical decisions. The decision to disclose this information to families or patients is also at the discretion of the medical staff. The surprise question was collected from nurses after patients had been admitted. Since 2016, Taipei City Hospital’s Emergency Department has systematically documented family meetings dedicated to discussions and decisions regarding Do Not Resuscitate (DNR) orders, including the formal signing of DNR permits.

As it is a retrospective study conducted in 2021 the committee of Institutional Review Board of Taipei City Hospital therefore approved a waiver for documenting informed consent due to the de-identified data.

### In-time and out-of-time validation sample design

As the aim of the present study is to develop the A-qCPR model to predict one-year mortality in ED patients, the A-qCPR model needs to be developed to identify independent variables (risk factors) of the past 12 months and death in the next 12 months in ED patients enrolled from the above-mentioned retrospective cohort. In addition, to enhance the robustness of the predictive model to accommodate different periods as mentioned above, we further divide the total sample of the retrospective cohort into an in-time validation sample and an out-of-time validation sample. The former is used for the development of A-qCR with the trained and holdout validation cohort from the Rei-Ai branch during the window period between 2015 and 2017. The latter is tailored for the external validation of the developed model to the out-of-time sample cohort during the period between 2017 and 2020, mainly from the emergency department of Taipei City Hospital, Zhongxiao branch between 2017 and 2020, and also from the Ren-Ai branch between 2018 and 2020 (Figure S2). A quasi-experimental design was used to evaluate the impact of an automated risk-scoring system incorporating the screening tool on the frequency and outcomes of family discussions regarding DNR decision-making and the signing of DNR permits in the Emergency Department (ED) of Taipei City Hospital.

### The modified A-qCPR model

The A-qCPR model was developed to predict 1-year mortality using five significant predictors: age, cancer presence, DNR status, a qSOFA score above two, and a Performance Status Score (PSS) above two. Following the incorporation of a new training cohort, there is a minor deviation in the risk scoring of the optimal model compared to the preliminary report, as delineated in the subsequent results (Table 1). The model assigns points as follows: age (0.05 points/year), qSOFA score > 2 (4 points), cancer (5 points), ECOG-PS > 2 (2 points), and DNR status (2 points). Patients are then categorized into low ( ≦ 3 points), intermediate (3–9 points), or high (> 9 points) risk levels [16]. The demographic, clinical outcome-related data, ECOG performance status score, and DNR status were also collected. In addition, the automated risk scoring system was established in the ED of five branches of Taipei City Hospital by the end of 2017. Therefore, we categorized emergency patients into high, medium, or low risk of death for the following year according to the risk score.

**Table 1:** Characteristics of patients in the training cohort and validation cohort

	Training cohort (n = 10,474)	Validation cohort a (n = 15,899)	Validation cohort b (n = 17,283)
**Age**			
mean (SD)	69.2 (20.0)	71.3 (16.4)	70.7 (17.2)
**Gender**			
Male	2,554 (50.4%)	8,245 (51.9%)	9,194 (53.2%)
Female	2,517 (49.6%)	7,654 (49.1%)	8,089 (46.8%)
**DNR**			
Yes	950 (18.7%)	3,406 (21.4%)	3,650 (21.1%)
No	4,121 (81.3%)	12,493 (78.6%)	13,633 (78.9%)
**Disease**			
Trauma	1,128 (10.8%)	3,441 (21.6%)	3,110 (18.0%)
Non-trauma	9,346 (89.2%)	12,458 (78.4%)	14,173 (82.0%)
Cancer	2,633 (25.1%)	3,145 (19.8%)	3,616 (20.9%)
Non-cancer	7,841 (74.9%)	12,754 (80.2%)	13,667 (79.1%)
**SQ**			1
Yes	8,148 (77.8%)	13,809 (86.8%)	3,158 (76.1%)
No	2,326 (22.2%)	2,090 (13.2%)	4,125 (23.9%)
**qSOFA**			
< 2	4,705 (92.8%)	14,935 (93.9%)	16,631 (96.2%)
≥ 2	366 (7.2%)	964 (6.1%)	652 (3.8%)
**PSS**			
≥ 2	1,609 (31.7%)	4,863 (30.6%)	5,846 (33.8%)
< 2	3,462 (68.3%)	11,036 (69.4%)	11,437 (66.2%)
**Death**			
Yes	3,556 (34.0%)	6,748 (42.4%)	6,890 (39.9%)
No	6,918 (66.1%)	9,151 (57.6%)	10,393 (50.1%)

Validation cohort a: Ren-Ai branch of Taipei City Hospital from 2018 to 2020

Validation cohort b: Zhongxiao branch of Taipei City Hospital from 2017 to 2020

DNR: Do-not-resuscitate, SD: standard deviation, SQ: Surprise Question when used by nurses

qSOFA: Quick Sequential Organ Failure Assessment; PSS: Eastern Cooperative Oncology Group Performance Status Score

### Statistical analysis

Chi-square test for categorical data, and T-test or ANOVA were used for continuous data. A two-sided *P* value of less than 0.05 indicated statistical significance. Odds ratios (OR) with 95% confidence intervals (CI) were calculated by logistic regression model. In addition, the multi-variable logistic regression model with a stepwise selection procedure (*P* for entry < 0.1; *P* to remove > 0.05) was used for identifying the most important determining factors for one-year mortality.

A score-based prediction model for the mortality was developed from logistic regression models using a regression coefficient-based scoring method. In addition, the optimal cutoff value was determined by Youden index. In-time validation with data between 2015 and 2017 was performed with 70% data used for training and 30% retained as holdout (test) sample. External validation was also performed by using out-of-time validation samples. The accuracy of the proposed model was evaluated by the area under the ROC curve (AUROC) for in-time and out-of-time validation. A summary ROC curve was also plotted based on the validated predicted probabilities.

Survival was estimated using the Kaplan-Meier method, and differences in survival between groups were assessed using the log-rank test. The population was divided into three categories according to the risk score calculated for each patient. The score was calculated to provide a tool for predicting survival. Survival curves were plotted to show the risk of death for both the in-time and out-of-time cohorts.

SAS statistical software (Version 9.4; SAS Institute Inc, Cary, NC) was used for these analyses. The study was approved by the Taipei City Hospital Institutional Review Board (TCHIRB-10,703,107, 13 April 2018; TCHIRB-11,008,011-E, 27 August 2021).

## Results

A total of 10,474 patients were enrolled for an in-time validation cohort from June 2015 to December 2017 in the Ren-Ai branch of Taipei City Hospital. Of these patients, 3,556 patients died during this study period. A total of 15,899 patients in the ED of Ren-Ai Branch from 2017 to 2020 and 17,283 patients in the ED of Zhongxiao Branch of Taipei City Hospital were enrolled for external validation based on the out-of-time validation sample. Of them, 13,638 (41.1%) patients died during the study period. Their baseline characteristics are shown in Table 2.

**Table 2:** Risk scores for 1-year mortality and the probability of a one-year mortality rate by risk category in the in-time cohort and out-of-time validation cohort

ED Risk Scores (In-time cohort)						
Risk Factor	Multivariable Adjusted OR (95% CI)		Beta Regression Coefficient		Points	
Age	1.02 (1.01–1.02)		0.016		0.05 / year	
qSOFA ≥ 2	2.96 (2.54–3.46)		1.09		4	
PSS ≥ 2	1.62 (1.45–1.82)		0.48		2	
Had DNR	1.98 (1.74–2.25)		0.68		2	
Had Cancer	4.31 (3.85–4.83)		1.46		5	
Probability of Mortality						
In-time cohort (*N* = 10,474)						
Risk Category		Cases n (%)		One-year mortality (95% CI)		
Low (score ≦ 3)		4165 (39.8%)		9.3% (8.4 − 10.2%)		
Intermediate (3 < Score ≦ 9)		4868 (46.5%)		23.7% (22.5 − 24.9%)		
High (score > 9)		1441 (13.8%)		44.3% (41.8 − 46.9%)		
Out-of-time validation cohort (*N* = 33,182)						
Risk Category		Cases n (%)		One-year mortality (95% CI)		
Low (score ≦ 3)		10,077 (3.4%)		13.6% (12.9-14.2%)		
Intermediate (3 < Score ≦ 9)		17,752 (53.5%)		29.9% (29.2-30.5%)		
High (score > 9)		5,353 (16.1%)		47.1% (45.8-48.4%)		

The risk category was calculated for each patient by adding together the points corresponding to his or her risk factors and defined three risk groups: low risk (score ≦ 3 points), intermediate risk (3 < Score ≦ 9 points), and high risk (Score > 9 points)

ED: emergency department; qSOFA: Quick Sequential Organ Failure Assessment; CI: confidence interval

PSS: ECOG (Eastern Cooperative Oncology Group) Performance Status Score; DNR: Do-not-resuscitate

**Fig. 1:**
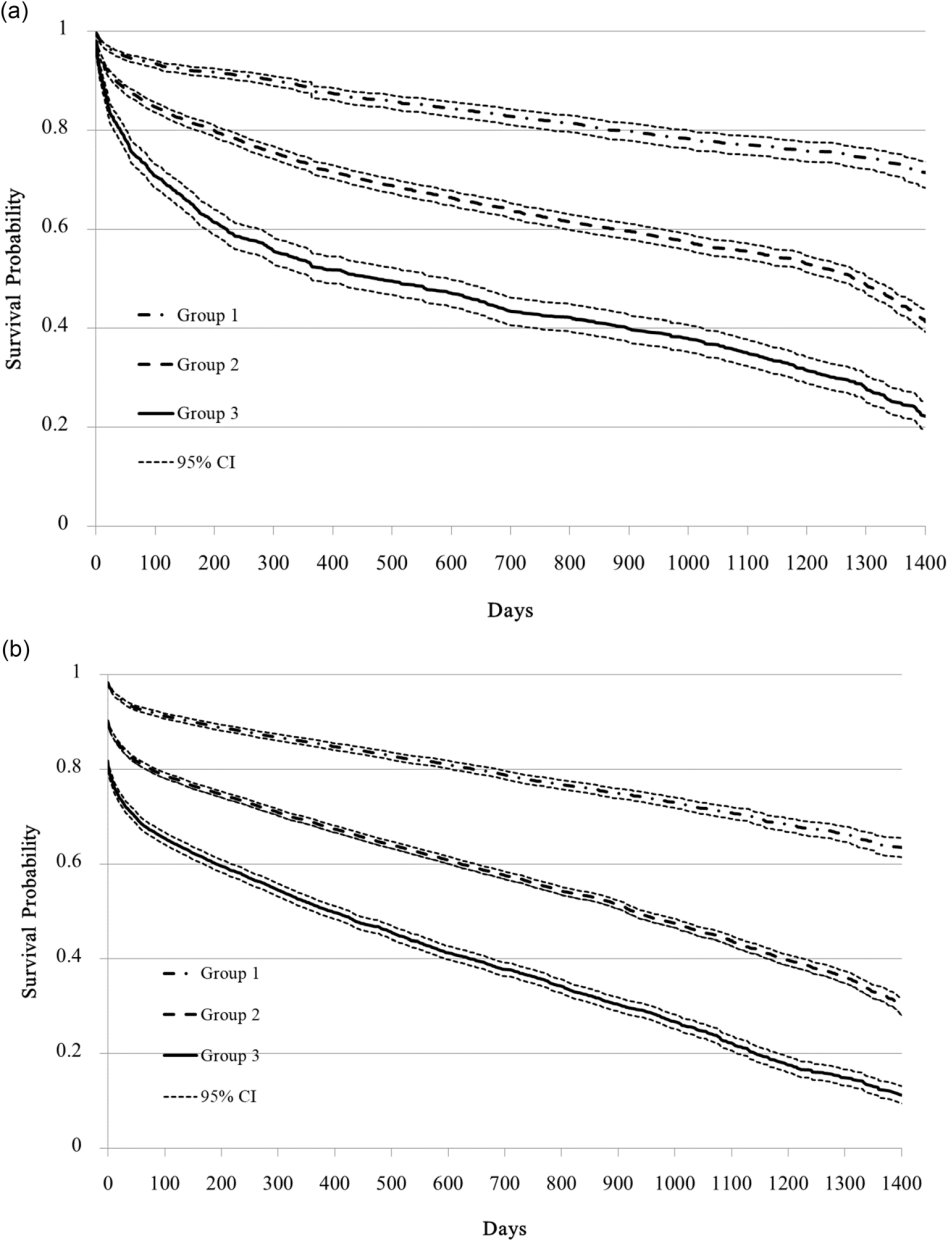
Kaplan-Meier survival estimates according to risk groups. **Group 1**: Risk scores ≦ 3; **Group 2**: 3 < Risk scores ≦ 9; **Group 3**: Risk scores > 9. [Logrank test *p* < 0.001; CI: confidence interval]. (**a**) Survival curves of admitted patients from the training cohort in the emergency department (*n* = 10,474). **b** Survival curves of those admitted patients from the validating cohort in the emergency department (*n* = 86,016)

In the in-time validation cohort, detailed risk scores and mortality probabilities can be found in Table 1. In the out-of-time validation cohort, the one-year and four-year mortality rates among the high (Score > 9 points), medium (3 < Score ≦ 9 points), and low-risk (score ≦ 3 points) groups are also shown in Table 1. The 1-year mortality rates in the training cohort are shown in Fig. 1a. The 1-year mortality rates in the out-of-time validation cohort for these three categories were 13.6%, 29.9%, and 47.1%, respectively (Table 3; Fig. 1b). The median follow-up time for patients still alive was 273 days (range, 0-1721 days) in the in-time validation cohort and 501 days (range, 0-1701 days) in the out-of-time validation cohort.

**Table 3:** The sensitivity, specificity, PPV, and NPV of screening tools for one-year mortality among the validation cohort in the emergency department

Screening Tools	Validation cohort	Sensitivity (95% CI)	Specificity (95% CI)	PPV (95% CI)	NPV (95% CI)
SQ	All	0.256	0.868	0.598	0.673
		(0.250–0.261)	(0.866–0.871)	(0.590–0.606)	(0.670–0.677)
PSS	All	0.474	0.700	0.336	0.807
		(0.468–0.481)	(0.697–0.703)	(0.330–0.341)	(0.803–0.810)
qSOFA	All	0.179	0.900	0.364	0.774
		(0.174–0.184)	(0.898–0.902)	(0.355–0.373)	(0.772–0.777)
A-qCPR model	All	0.918	0.214	0.273	0.891
(score > 3 points)		(0.915–0.922)	(0.211–0.217)	(0.270–0.276)	(0.886–0.895)

SQ: Surprise Question; qSOFA: Quick Sequential Organ Failure Assessment

PSS: ECOG (Eastern Cooperative Oncology Group) Performance Status Score

PPV: positive predictive value; NPV: negative predictive value

CI: Confidence Interval

A-qCPR model: the model incorporates factors such as **A**ge, **q**SOFA, **C**ancer, **P**erformance status scale, and DN**R** (Do-not-resuscitate)

**Fig. 2:**
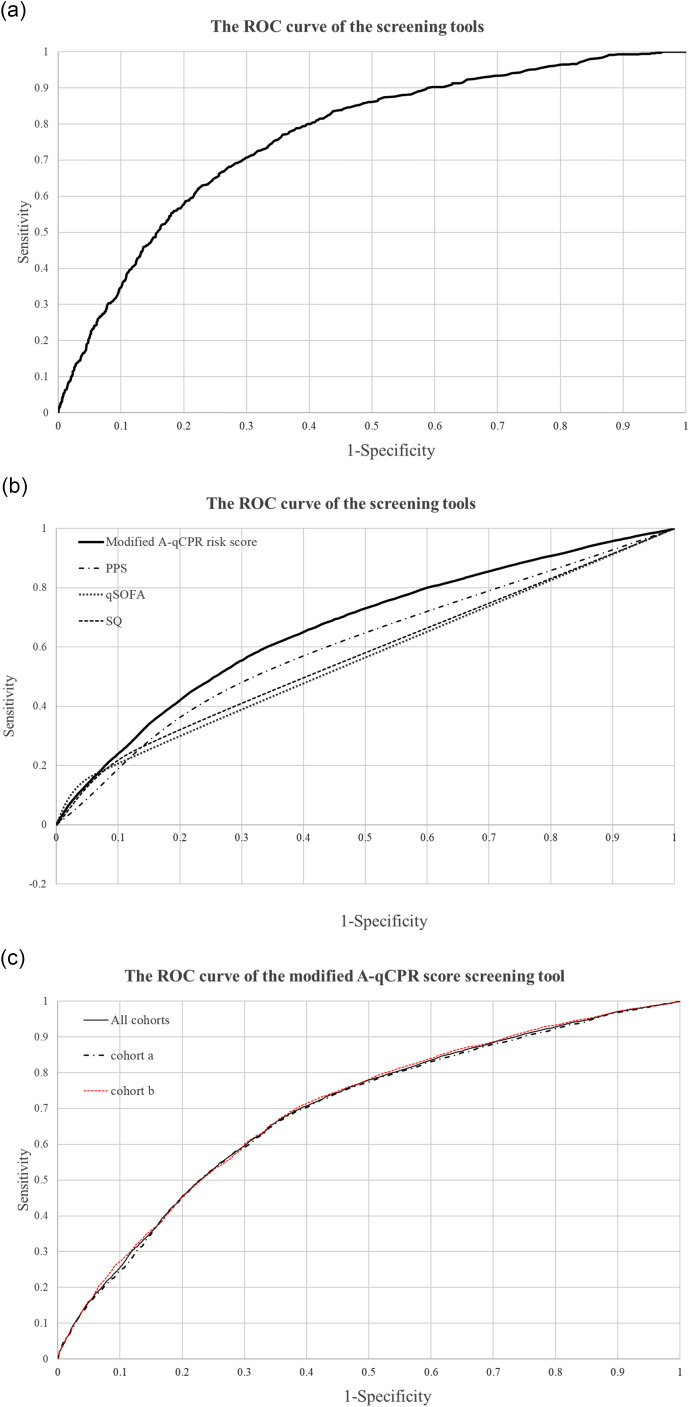
Receiver Operating Characteristic Curve for 1-year mortality among admitted patients in the emergency department. **a** The area under the ROC curve (AUROC) is 0.762 (0.742–0.782) for the A-qCPR risk score in the training cohort with cross-validation. **b** The area under the ROC curve from the out-of-time validation sample is 0.694 (0.688-0.700) for the A-qCPR risk score, 0.590 (0.587–0.594) for PPS, 0.539 (0.536–0.542) for qSOFA, and 0.565 (0.562–0.568) for SQ. [PPS: Eastern Cooperative Oncology Group Performance Status Score; qSOFA: Quick Sequential Organ Failure Assessment; SQ: Surprise Question]. **c** ROC curves for 1-year mortality based on the training cohort validated predicted probabilities for the A-qCPR screening tool, the area under the ROC curve is 0.694 (0.688–0.701) in the out-of-time validation sample, and it is 0.691 (0.682-0.700) and 0.698 (0.689–0.707) in the validation cohort a and b, respectively

The area under the ROC curves for the modified A-qCPR model were 0.745 (0.732–0.759) for the training data and 0.762 (0.742–0.782) for the holdout data (Fig. 2a). The optimal cutoff point of scores in our model was 3 by the Youden index in the training cohort. However, the area under the ROC curve for out-of-time validation sample was 0.694 (0.688-0.700) (Fig. 2b). In addition, the misclassification rate of our model for 1-year mortality was 20.8% for the out-of-time validation sample. However, the area under the ROC curve for 1-year mortality was 0.565 (0.562–0.568) by using SQ, 0.539 (0.536–0.542) by using qSOFA, and 0.590 (0.587–0.594) by using ECOG performance status score (Fig. 2b). The area under the curve for 1-year mortality by using the A-qCPR screening tool was 0.694 in the out-of-time validation sample, and 0.691 and 0.698 in validation cohorts a and b, respectively (Fig. 2c).

The sensitivity, specificity, positive predictive value (PPV), and negative predictive value (NPV) of screening tools (i.e. SQ, PSS, qSOFA, and A-qCPR model) are shown in Table 3. The optimal cutoff value was determined using the Youden index in our model. Our risk score screening tool for 1-year mortality had high sensitivity of 0.918 ( 0.915–0.922) and a high NPV of 0.891 ( 0.886–0.895). The ECOG performance status score for 1-year mortality had also high NPV 0.807 (0.803–0.810) The SQ and qSOFA for 1-year mortality had high specificity. But the tools of SQ, ECOG performance score, and qSOFA had lower sensitivity than the A-qCPR risk score.

**Fig. 3:**
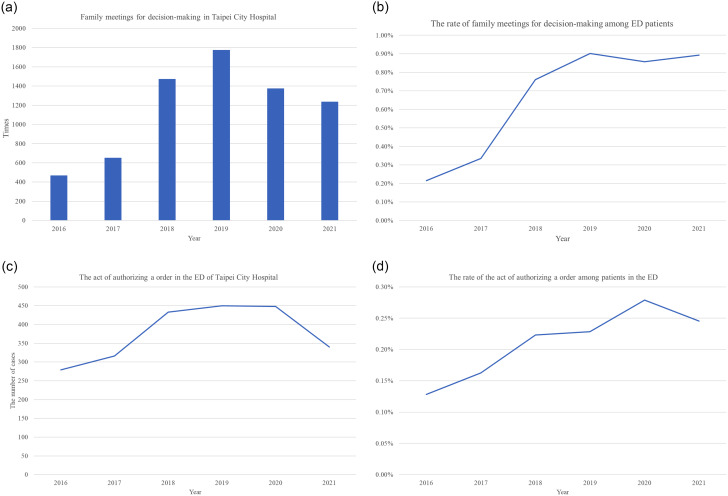
Family meetings for the DNR decision-making and signing the DNR (Do-Not-Resuscitate) permits in the ED (emergency department) of Taipei City Hospital from 2016 to 2021. **a** Family meetings for the DNR decision-making process. **b** The rate of family meetings for decision-making among patients in the ED. **c** The act of authorizing a DNR order. **d** The rate of the act of authorizing a DNR order among patients in the ED

Figure 3a shows the number of family meetings held in the emergency department. After the automated risk scoring system was established at the end of 2017, the frequency of family meetings held in emergency departments increased significantly. The ratio of the number of family meetings to the number of emergency department patients has been on the increase year by year. (Fig. 3b). However, their frequency decreased significantly due to the impact of the COVID-19 epidemic after 2020. The number of patients who signed the authorizing DNR order form is shown in Fig. 3c. There was also a year-on-year increase in the ratio of DNR signatories to emergency department patients (Fig. 3d). They have not been affected by the epidemic of COVID 19.

## Discussion

The scores of the A-qCPR model allow the clinician to identify a higher risk of mortality within one year in the emergency department. Our screening tool has a low misclassification rate, higher sensitivity, and NPV. This information might be used to select patients for the need of hospice in the emergency department. As each item can be easily collected, our screening tool can be easily accessed in an emergency or critical care setting.

Patients with scores > 3 had higher risk mortality within one year, and they could potentially benefit the most from being referred to specialist palliative consultations or aggressive therapies. Our primary focus is to prioritize the hospice and PC needs of high-risk patients in the emergency department. At the end of 2017, an automated risk scoring system was implemented in the emergency department of Taipei City Hospital according to the preliminary report [16]. After patient triage, the system stratified all ED patients into three groups based on our risk score. After patient triage, the system stratified all ED patients into three groups based on our old risk score. The implementation of the automated scoring system alerts emergency physicians, nurses, patients, and their families to the risk of mortality during the next year and hospitalization, facilitating the process of decision-making regarding DNR documents. This is supported by the fact that the rate of patients or their families signing DNR documents has increased every year, and this trend has not been affected by the COVID-19 pandemic. We believed that the prognostic information from our model could remind emergency physicians of the need for hospice care for patients, and help those patients and their families make timely decisions. Notably, even during the COVID-19 pandemic, when organizing family meetings was a challenge, the rate of patients deciding on DNR orders in the emergency department still increased.

The revised A-qCPR model introduces changes in scoring for qSOFA, cancer, and DNR status, adopting a more conservative threshold for low-risk categorization (≤ 3 points). It outperforms the original model in discriminative ability during in-time validation (AUROC of 0.76 vs. 0.707), indicating improved predictive accuracy for one-year mortality. However, a decline in AUROC to 0.69 in out-of-time validation highlights concerns about its generalizability and reliability over time, emphasizing the need for ongoing evaluation and adjustment to maintain its effectiveness across different patient cohorts and temporal settings. The updated version of the risk scoring system mainly increased the weight of qSOFA and cancer, while decreasing the weight of DNR. In the early stages of this study, many terminal-stage patients visited our ED for help, which may imply that hospice was not provided or too late. The number of patients mentioned above decreased in our ED after hospice care was promoted in Taipei City Hospital. Hence, a patient who signed the authorizing DNR document was strongly correlated with the risk of death in the early stage. Furthermore, as hospice with PC education and training for healthcare providers have been progressively implemented, earlier communication with patients and their families facilitates earlier provision of hospice care. As a result, the strength of the correlation between a patient signing the authorizing DNR order and the risk of death has decreased somewhat. The weight of DNR was attenuated in the updated model. The area under the ROC curves for the model was 0.84 (0.83–0.85) in our preliminary report [16]. Though the accuracy of predicting 1-year mortality decreased in the validation cohort, the advantage of our screening tool had a low misclassification rate, higher sensitivity, and NPV. For hospice decision-making, the A-qCPR model’s high sensitivity is crucial for accurately identifying patients who could benefit from end-of-life care, despite its low specificity, which leads to more false positives. In clinical practice, the model’s utility lies in ensuring no eligible patient is overlooked for hospice care, with the trade-off being the need for careful secondary evaluation to manage the implications of its false positive rate within the context of resource allocation and patient-centered care considerations.

However, the use of the screening tool can facilitate the process of making EOL decisions. The unnecessary and non-beneficial invasive interventions might be decreased by using such an advanced screening tool for hospice needs. The frequency of family meetings in emergency departments increased markedly over the years, although there was a significant decrease after 2020 due to the COVID-19 pandemic. Conversely, the proportion of emergency patients signing DNR orders increased steadily and was not affected by the epidemic. Hence, hospitals may be culturally motivated to promote high-intensity EOL care following the use of the hospice screening tool and EOL decision-making process. One study reveals that hospitals inherently favor high-intensity EOL care due to prevailing cultures and institutional structures. The efficacy of individual efforts can be undermined by these cultures and inadequate supportive policies, emphasizing the need to consider hospital culture when devising interventions against potentially unnecessary, intensive treatments [17]. 

It is difficult for emergency physicians and nurses to predict that a patient will die within the next six months, and even more challenging to predict that a patient will die the next year. The accuracy of the SQ was varied in previous reports [12]. In addition, the accuracy and sensitivity of using the SQ to predict mortality were very low in our study. While previous research has established the prognostic value of the ECOG PS score in critically ill patients, [18] our study found that it was not a reliable screening tool for ED patients. The qSOFA was good for predicting in-hospital mortality for patients with suspected infection in the emergency department [19]. In addition, the prognostic performance of the Emergency Severity Index (Triage scale) with qSOFA for in-hospital mortality was high (AUROC = 0.79) in the previous study [20, 21]. However, The Emergency Severity Index accurately predicted high dependency unit/ICU admissions and 3-day mortality for patients ≥ 65 years but was inaccurate for 30-day mortality and hospital admissions across both age groups [22]. Although 1-year mortality was highly associated with qSOFA in our study, it was not a good screening tool for hospice needs due to low sensitivity and accuracy of prediction. Note that the Emergency Severity Index was not associated with one-year mortality in our training cohort and it was not significantly associated with 1-year mortality in our final model.

DNR status might be associated with a decrease in critical interventions and procedures, worse prognosis, higher comorbidity burden, and high mortality among patients with sepsis, and serious trauma, following resuscitation from out-of-hospital cardiac arrest [23–28]. Hence, DNR status associated with higher 1-year mortality rates was also noted in our study.

### Limitations

This study had several limitations. Despite the improvement in the accuracy of the previous prediction model with the modified A-qCPR model proposed here, there is still room for improvement. In this study, we did not consider the influence of therapeutic interventions, disease severity, biomarkers, vital signs or diagnoses. Further research could integrate all the potential determinants of mortality mentioned above and use machine learning techniques to build a more delicate model to improve the accuracy of mortality prediction. However, our risk assessment score is easy to calculate and implement, making it suitable as a screening tool. Second, the results of this study may not be generalizable to patients in hospital emergency departments in different regions and countries. This is because different regions or countries may have different disease burdens and populations with different genetic susceptibility, demographics, clinical features, and health behaviors. They also have a unique healthcare system with different standards, practices, resources, and treatment guidelines. This variation may affect the generalizability of the results. However, the proposed methodology and design can be still adapted to develop the adequate predicted model on their own. Such a study is very difficult to have a control group because of ethical considerations. Moreover, as our main objective of this study is to advance an already existing screening tool such as the A-qCR model for hospice care, the effectiveness of the increased signed DNR study is therefore not the primary objective. It would be of great interest to design an ongoing study for such a purpose. Therefore, it is difficult for our study to elucidate the effect of our automated risk-scoring system on DNR decision-making at Taipei City Hospital, which limits the ability to definitively attribute changes in DNR discussions and decisions to the screening tool (automated risk-scoring system).

## Conclusion

Our risk score can be used for 1-year mortality prediction and screening for the hospices need. Real-time implementation of risk scores can be auto-calculated in our HIS (Hospital information system) system after the triage registry in our emergency department. The use of updated risk scores should be considered to improve predictive accuracy by incorporating more variables, such as blood test results. Further research is needed to assess the effectiveness of using this screening tool to improve the quality of decision-making in emergency departments.

### Electronic supplementary material

Below is the link to the electronic supplementary material.

**Supplementary Material 1:** Descriptive data of Taipei City Hospital's Branches and the Original and Modified Risk Scores

## Data Availability

Data analyzed during this study are available from the corresponding author upon reasonable request.

## Abbreviations

EOL: End-of-life
PC: Palliative care
ED: emergency department
qSOFA: Quick Sepsis Related Organ Failure Assessment
DNR: Do-Not-Resuscitate
A-qCPR: Age, qSOFA, cancer, Performance Status Scale, and DNR
SQ: surprise question
ECOG: Eastern Cooperative Oncology Group
AUROC: The area under the receiver operating characteristic curve
