# Supplementary material for: Advancing screening tool for hospice needs and end-of-life decision-making process in the emergency department
Source: BMC Palliat Care. 2024 Feb 23;23:51. doi: 10.1186/s12904-024-01391-w (PMC10885365; doi:10.1186/s12904-024-01391-w)
Supplement: Supplementary file 1 — Supplementary Material 1: Descriptive data of Taipei City Hospital's Branches and the Original and Modified Risk Scores [file 12904_2024_1391_MOESM1_ESM.docx]

***Supplementary:* Advancing Screening Tool for Hospice Needs and End-of-Life Decision-making Process in the ED (emergency department)**

**Taipei City Hospital**

Taipei City Hospital in Taipei, Taiwan, is a multi-faceted healthcare organization with several branches scattered throughout the city. The locations of Taipei City Hospital's branches are shown in Figure S1. The general medical branches of the hospital include Heping Fuyou, Renai, Zhongxing, Zhongxiao, and Yangming. These branches provide a wide range of medical services, including internal medicine, surgery, pediatrics, obstetrics and gynecology, neurology, rehabilitation, cardiovascular surgery, organ transplantation, emergency medicine, intensive care, geriatric care, and palliative care. In addition, the special medical branches include the Songde Branch for psychiatric care, the Linsen Branch for traditional Chinese medicine care, and the Kunming Branch for chronic infectious disease management and drug addiction prevention and treatment. All ED patients were collected from general branches of Taipei City Hospital in this study. The relevant data from Taipei City Hospital are shown in Table S1.

**The original risk score and the validation for one-year mortality**

Risk scores were developed in our previous study [1]: age (0.05 per year), qSOFA ≥ 2 (1 point), Cancer (4 points), EOCG Performance Status score ≥ 2 (2 points), and DNR status (3 points). The AUROC curve for our model was 0.707 (0.700-0.714) based on the all validation cohort. The 1-year-mortality rates for these three categories, low risk (≦4 points), intermediate risk (between 4 and 9), and high risk (more than 9 points), were 12.5% (12.2%-12.9), 26.8% (16.4%-27.2%), and 43.1% (42.2%-43.9%) in the validation cohort, respectively.

**The modified risk score and the validation for one-year**

Patients’ admission age, cancer, DNR status, qSOFA of more than two, and PSS (Performance Status Score) of more than two were demonstrated the significant predictors for 1-year mortality in the training cohort. Hence, the A-qCPR model was developed from the training cohort: Age (0.05 points per year), qSOFA more than two (4 points), evidence of Cancer (5 points), ECOG Performance status score (ECOG-PS) more than two (2 points), and DNR status (2 points). Personalized risk scores were calculated for each patient and defined three risk categories: low risk (≦3 points), intermediate risk (between 3 and 9), and high risk (> 9 points). Risk scores and the probabilities of 1-year mortality in the training cohort are listed in Table 2. Therefore, the logistic regression model equation can then be written as:

$\text{L}og\begin{matrix} Odds=\text{L}og\left(\frac{p}{1-p}\right)-3.262+0.016X_{1}+1.09X_{2}+0.48X_{3}+0.68X_{4}+1.46X_{5} & \end{matrix}$ .

Where X_1-5_ denotes Age, qSOFA, ECOG-PS, DNR status, and Cancer respectively.

Table S1. Taipei City Hospital profiles

|  | A branch | B branch | C branch | D branch | E branch | Total |
| --- | --- | --- | --- | --- | --- | --- |
| Acute care beds | 351 | 296 | 237 | 238 | 263 | 1,385 |
| ICU beds | 30 | 30 | 24 | 25 | 19 | 128 |
| Annual ED visits, 2017 ~2020  Mean (SD) | 35,681 (2,982.9) | 55,735 (7,006.5) | 41,344 (3,978.6) | 22,768 (1,856.0) | 28,814 (2,573.7) | 55,735  (7006.5) |

A branch: Renai branch, Taipei City Hospital; B branch: Heping Fuyou branch, Taipei City Hospital; C branch: Zhongxiao branch, Taipei City Hospital; D branch: Yangming branch, Taipei City Hospital; E branch: Zhongxing branch, Taipei City Hospital.

ICU: Intensive Care Unit; ED: emergency department; SD: Standard deviation.

**Figure S1. The locations of the branches of Taipei City Hospital**

**
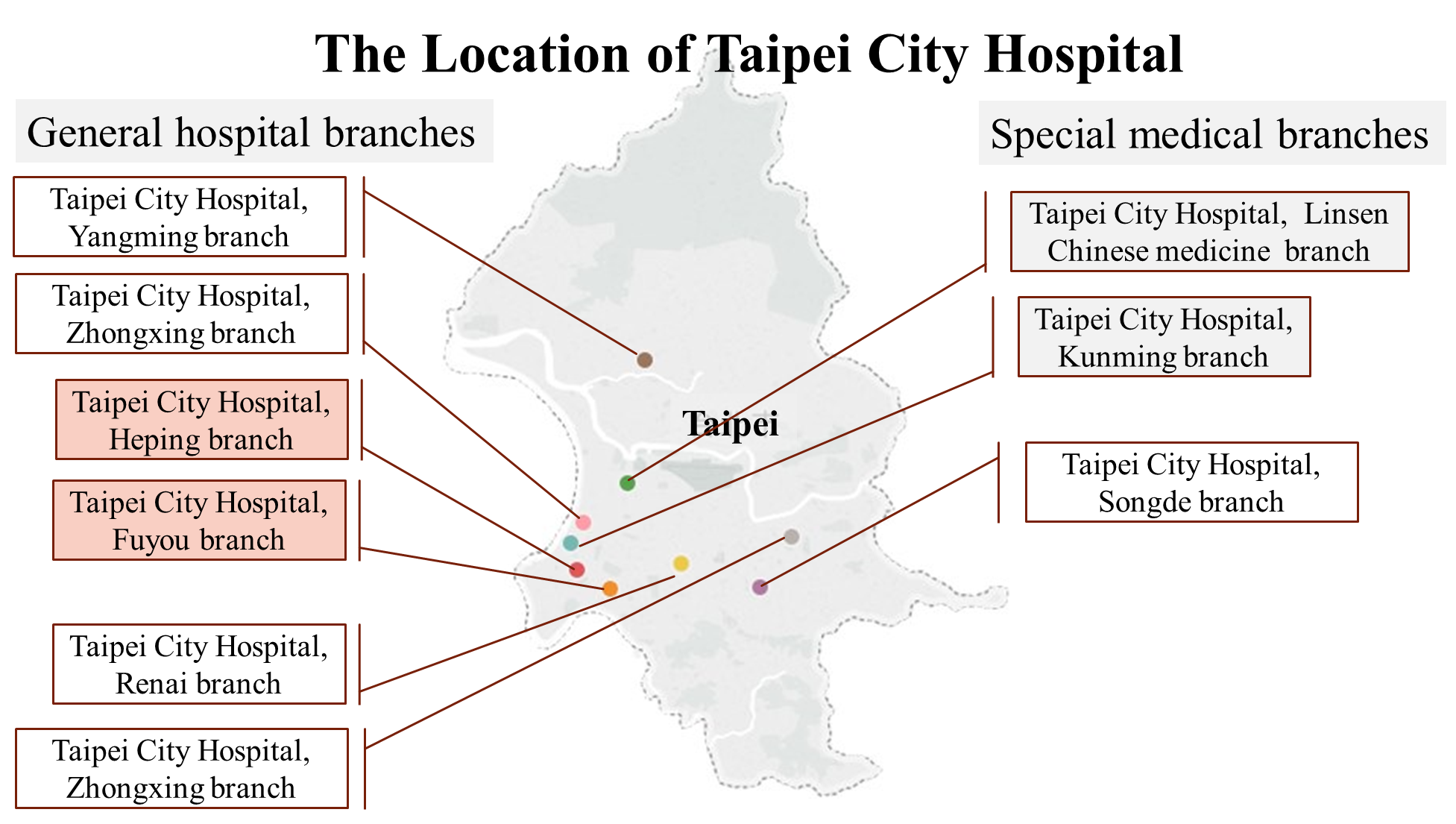
**

**Figure S2. Illustration of In-time and Out-of-time Validation Sample Design**

**
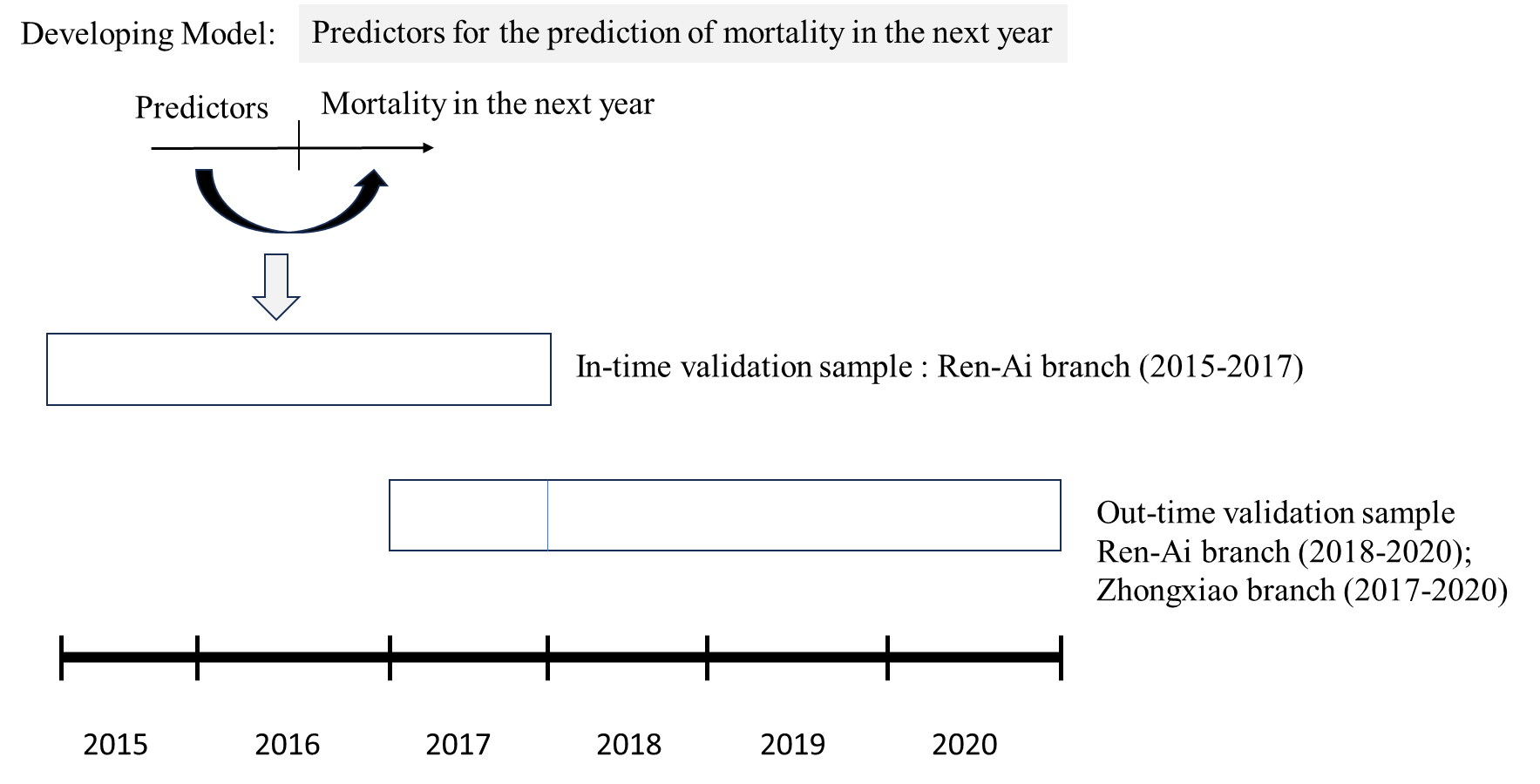
**
